# Canine dermatophytic kerion caused by *Microsporum canis*: two case reports

**DOI:** 10.3389/fvets.2026.1809436

**Published:** 2026-08-11

**Authors:** Ailén Dumont-Viollaz, Belén Rivera Gómez-Barris, Leslye Camila del Río, Claudio Moreno, Pamela Thomson

**Affiliations:** 1 Faculty of Life Sciences, One Health Institute, Universidad Andres Bello, Santiago, Chile; 2 Facultad de Ciencias de la Vida, Escuela de Medicina Veterinaria, Universidad Andrés Bello, Santiago, Chile; 3 Programa de Doctorado en Medicina de la Conservación, Universidad Andrés Bello, Santiago, Chile; 4 Clínica Veterinaria Oftaderm, La Reina, Chile

**Keywords:** case reports, dermatophytosis, dog, kerion, *Microsporum canis*, zoonosis

## Abstract

Dermatophytosis is a superficial, highly contagious mycosis caused mainly by keratinophilic fungi of the genera *Microsporum, Trichophyton* and *Nannizzia*. This report describes two clinical cases of dermatophytic kerion in dogs caused by *Microsporum canis*. In both, direct microscopic examination of hairs revealed ectothrix arthroconidia, and fungal culture yielded colonies morphologically consistent with *M. canis*. Species identification was confirmed by PCR amplification and partial sequencing of the ribosomal ITS region, followed by BLAST analysis and phylogenetic reconstruction. Antifungal susceptibility testing was performed by agar diffusion. Systemic antifungal therapy using pulse regimens of itraconazole or terbinafine, combined with topical treatment and environmental decontamination, resulted in complete clinical and mycological cure in both patients. These cases emphasize the importance of considering dermatophytosis, particularly kerion, in the differential diagnosis of deep inflammatory skin lesions in dogs. Early recognition and appropriate antifungal therapy are essential to achieve successful outcomes and to reduce the risk of zoonotic transmission.

## Introduction

Dermatophytosis is a highly contagious superficial mycosis caused by keratinophilic fungi, commonly belonging to the genera *Microsporum, Trichophyton*, and *Nannizzia* (1). Particularly, *Microsporum canis* is a zoophilic dermatophyte with a wide geographic distribution and is one of the main etiological agents of dermatophytosis in dogs and cats, capable of establishing clinically evident infection in other mammals and humans (2–5). Clinically, in dogs, it manifests as circular, scaly, alopecic lesions, brittle hair, and varying degrees of erythema and pruritus (2). However, under certain circumstances, the host’s immune response can trigger atypical or more severe clinical presentations, among which kerion stands out. Kerion is a deep inflammatory form characterized by painful, exudative, edematous plaques or nodules with pustules, crusts, and marked alopecia (6), which is like inflammatory tinea capitis or kerion Celsi in humans (4, 7). This presentation can simulate other dermatoses of bacterial origin, parasitic processes, or even tumorous lesions, leading to diagnostic delays and inappropriate treatments, particularly when it is interpreted as a primary bacterial infection and antibiotic therapy is initiated without mycological confirmation (8, 9).

Dermatophytic kerion presents a significant clinical challenge, characterized by an intense inflammatory reaction caused by dermatophyte invasion of hair follicles and the dermis, triggering a severe T-cell-mediated hypersensitivity reaction (10, 11). Although historically associated with *Trichophyton* spp., it can also be caused by *M. canis*, highlighting its importance in the context of animals in close contact with humans (12). Therefore, etiological confirmation through mycological culture, microscopic evaluation, and complementary tests is essential for therapeutic management and the implementation of environmental control measures.

Here we describe two clinical cases of kerion in dogs caused by *M. canis,* emphasizing the dermatological findings, the diagnostic approach, and the relevance of early recognition of this inflammatory presentation, both to optimize antifungal treatment and to reduce the risk of dissemination and zoonotic transmission.

### Case descriptions

Case 1: On February 10, 2025 (Day 0), a 5-year-old female Silky Terrier was presented for consultation. She had a history of chronic pruritus (PVAS 6/10) (13) and multifocal alopecia, unresponsive to previous topical 2% chlorhexidine therapy (DifemPharma, Santiago, Chile) for 1 month. Her annual vaccinations and internal and external parasite control were up to date, and previous examinations performed during the consultation ruled out endocrinopathies as the underlying cause of immunosuppression.

**Figure 1:**
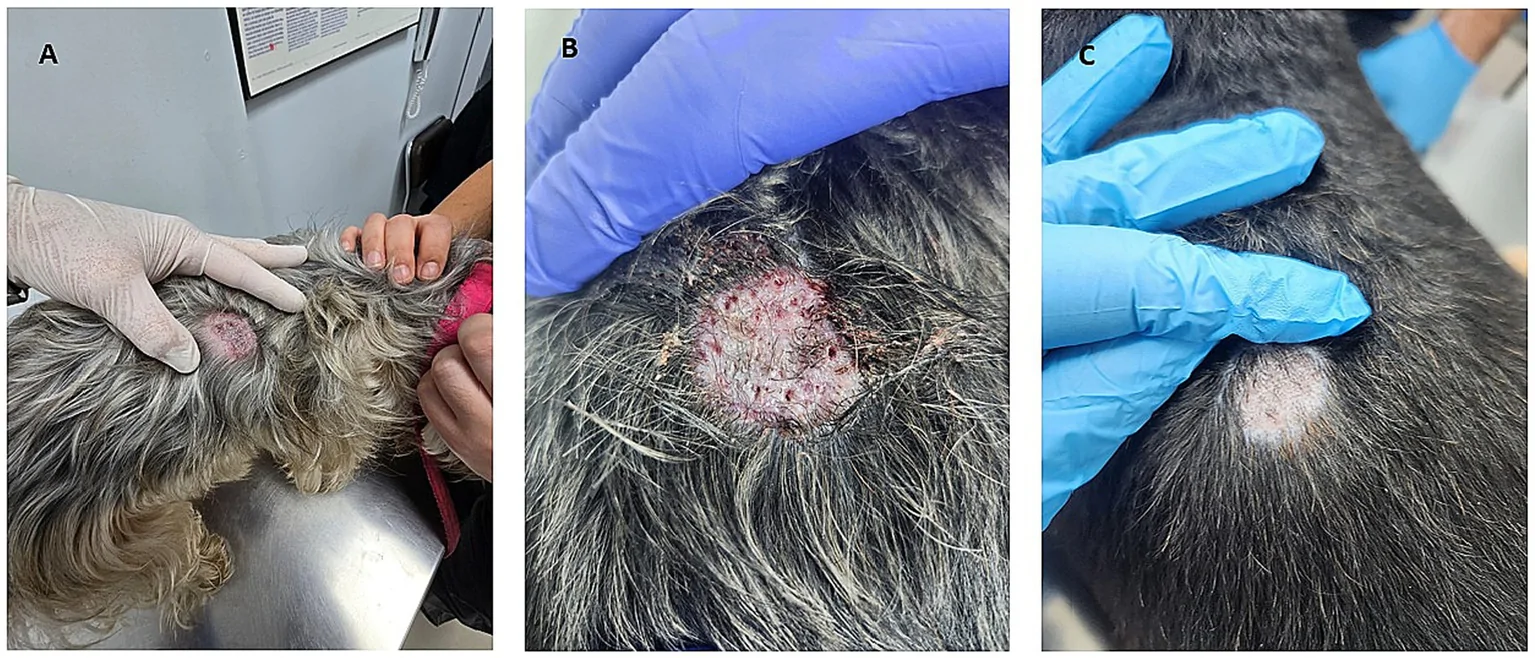
**(A,B)** A lesion on the back of a Silky Terrier, approximately 3 cm in diameter characterized by alopecia, inflammation, and small pustules. **(C)** No lesion observed on day +81. The hair is still in the growth process. The rest of the coat appears short after a visit to the groomer.

The dermatological clinical examination revealed a multifocal pattern with a dorsal distribution and affecting all four limbs. The lesions appeared as raised, alopecic, hyperpigmented, scaly, and erythematous plaques, 2–3 cm in diameter. Some plaques had draining tracts with serosanguineous exudate (Figures 1A,B). Hypotrichosis was also present in the abdominal area. Scabies and/or bacterial dermatitis were considered in the differential diagnosis.

Direct microscopic examination of the hair, performed during the dermatology consultation, showed arthroconidia in the examined hairs. Cytological examination revealed macrophages, abundant neutrophils, cocci bacteria (1/field), and evident free fungal structures. Samples were sent to the laboratory that same day for culture and antifungal susceptibility testing. While awaiting the results, the following treatments were prescribed: baths with miconazole and chlorhexidine shampoo twice a week (Drag Pharma, Quilicura, Chile) and topical clotrimazole 1% twice a day (Laboratorios Chile, Ñuñoa, Chile) for 21 days. The presence of 1 coccal bacteria/field was not considered for the prescription of systemic antibiotics.

**Figure 2:**
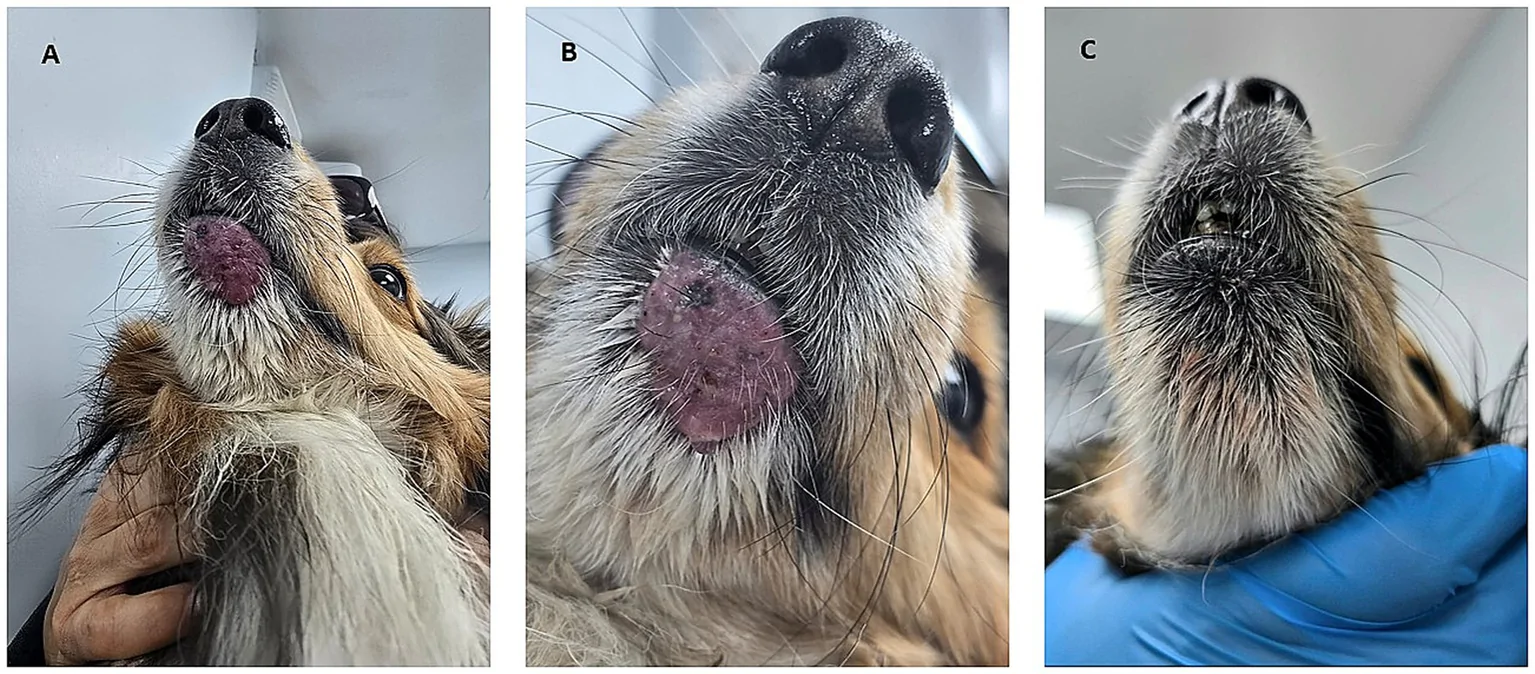
**(A,B)** Single alopecic lesion under the chin of 3.5 cm in diameter in a Shetland Sheepdog, consistent with a well-defined, erythematous-violaceous, irregular, slightly raised surface, with punctate hemorrhagic crusts and areas of superficial erosion. **(C)** Absence of the injury observed on day +60.

Case 2: On August 25, 2025 (day 0), an 8-year-old male Shetland Sheepdog was presented for consultation with a single lesion on his chin, which had appeared approximately 20 days prior (Figures 2A,B). The owner reported that she and two other dogs living in the same house began developing similar circular, erythematous lesions after adopting a cat with a presumptive diagnosis of dermatophytosis. The owner had been clinically diagnosed by human healthcare professionals and treated with a topical antifungal for a single lesion on her chest, with a good response to treatment.

The owner reported that the dog’s lesion had grown significantly after a previous 15-day course of corticosteroids and antibiotics. The dermatological examination revealed a circumscribed, alopecic lesion on the chin, 3.5 cm in diameter, slightly raised, inflamed, and shiny. In this case, dermatophytosis was immediately suspected. A Wood’s lamp examination was performed, revealing positive fluorescence, which allowed for the selection of affected hairs and scales for sampling. Trichogram analysis revealed abundant hairs with arthroconidia, and cytology showed coccoid bacteria (1-3/field). Pending mycological results, the area was cleaned with saline solution (Prontosan™, BBraun, Barcelona, Spain), followed by topical application of 2% mupirocin (Lazar, Buenos Aires, Argentina) twice daily and oral terbinafine 30 mg/kg once daily in pulse therapy, alternating 14 days of treatment with 7 days of rest in successive cycles. The presence of 1–3 coccal bacteria/field was not considered for the prescription of systemic antibiotics.

**Figure 3:**
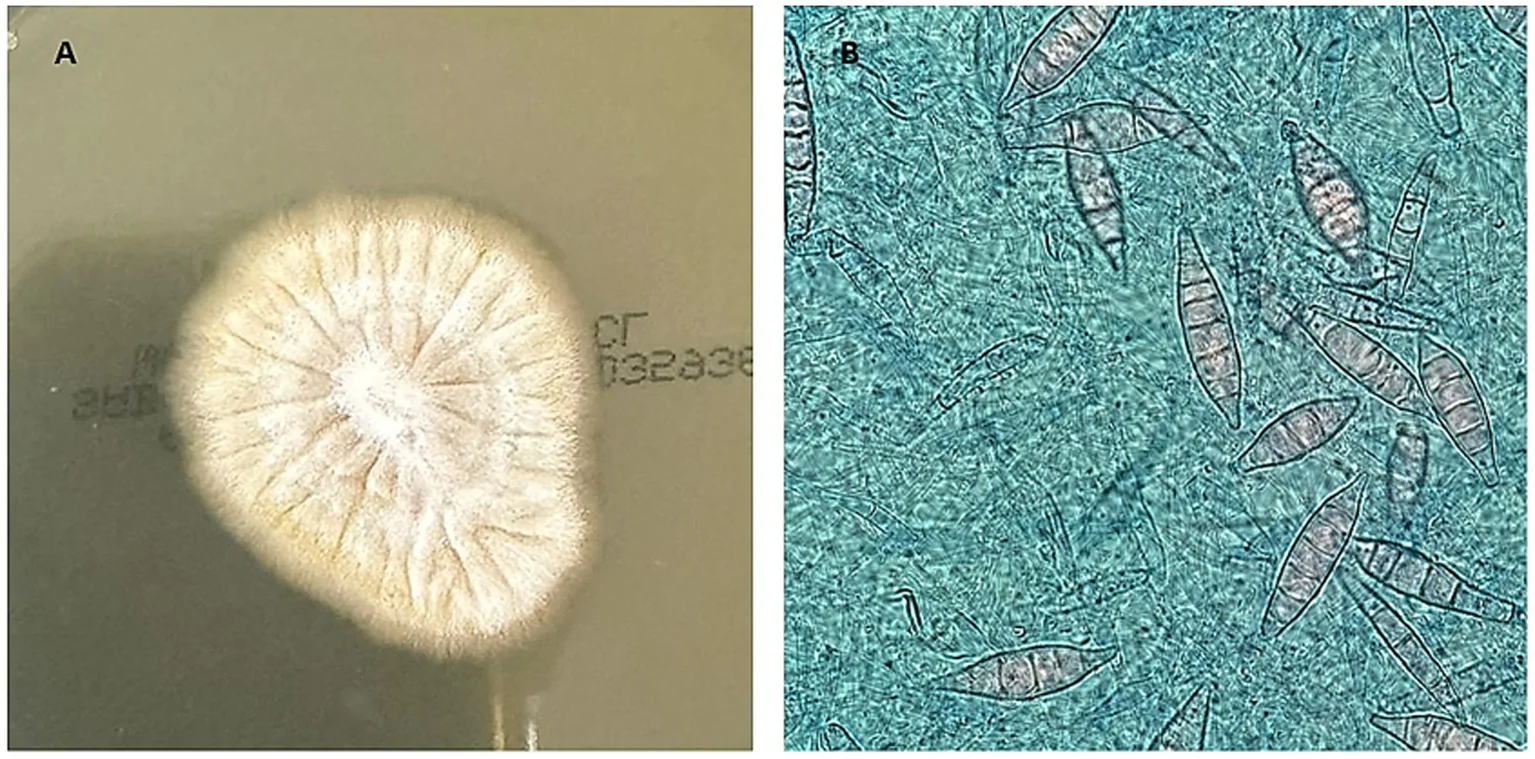
Macroscopic and microscopic appearance of *Microsporum canis*. **(A)** Colony after 20 days of growth on Sabouraud dextrose agar, with a cottony/woolly appearance, white to creamy-white surface, with a marked radial pattern, well-defined concentric folds, and furrows. The center is slightly raised, with well-defined, regular edges. **(B)** Microscopic view at 100× magnification, prepared with lactophenol cotton blue; abundant, fusiform to ellipsoidal macroconidia are observed, with thick, rough walls and tapered ends, exhibiting 6–15 transverse septa. Hyaline, septate hyphae are visible in the background of the microscopic field.

Diagnosis: Hair and scale samples obtained from the active lesions of both patients were received for microbiological analysis by the Clinical Microbiology and Microbiome Laboratory of Andrés Bello University. A direct examination of hair and scales was performed using 10% potassium hydroxide (KOH), which allowed visualization of arthroconidia in ectothrix on the examined hairs. Subsequently, the samples from both patients were cultured on Sabouraud dextrose agar with chloramphenicol and cycloheximide and incubated at 25 °C for 21 days. Macroscopically, the colonies were observed to be thin, dense, cottony, and with radiating edges, ranging in color from grayish to yellowish-white with an intense ochre-yellow reverse. Microscopic visualization with lactophenol cotton blue (Figure 3) showed septate hyaline hyphae giving rise to fusiform, verrucose, thick-walled macroconidia containing multiple cells (14). DNA extraction from a fresh colony was performed using the Quick-DNA Fungal/Bacterial Miniprep Kit (ZYMO Research, Irvine, CA, USA), and DNA quantification was performed using a Nanodrop 2000c nano spectrophotometer (ThermoScientific, Waltham, MA, USA). Subsequently, 10 ng of the extracted DNA was used for PCR amplification of the core region of the ribosomal internal transcribed spacer (ITS), using primers ITS4 5′-TCCTCCGCTTATTGATATGC-3′ and ITS5 5′-GGAAGTAAAAGTCGTAACAAGG-3′ (15). PCR amplification and Sanger sequencing were performed by a specialized external service. The sequencing of the ITS region encompassed the ITS1 (partial), the 5.8S ribosomal gene (complete) and ITS2 (partial) regions. The sequences obtained from the amplified regions were compared with those available in GenBank using the basic local alignment search tool (BLAST). The analysis showed 98.81% nucleotide identity and 100% query cover to *M. canis* isolate (GenBank accession number OW988272.1) for the first patient (GenBank accession number PV918591), and 100% nucleotide identity and 88% query cover to *M. canis* voucher SMUDH2299 (GenBank accession number PX049120.1) for the second patient (GenBank accession number PX458008), thus confirming the presence of *M. canis* in both patients.

A phylogenetic analysis was performed based on the obtained ITS sequences, the genetic distance was calculated, and a neighbor joining tree was created using MEGA version 11. For this purpose, (1) the ITS sequences obtained in this study (PV918591 and PX458008), and (2) freely available *M. canis* ITS sequences obtained from Genbank. As no prior Chilean *M. canis* ITS sequences were available for comparison, we used a comprehensive global dataset from Zhou et al. (4) to contextualize our isolates. Information for each sequence is available in Supplementary Table 1. The neighbor joining tree and the sequence location map were visualized using Microreact version 282 (Supplementary Figure 1). The phylogenetic analysis did not suggest clustering by host or geographical location.

In parallel, antifungal susceptibility was determined using the agar diffusion method (16), on Müller–Hinton agar without supplements, following the Clinical and Laboratory Standards Institute M51A guidelines (17). A conidial suspension corresponding to the 0.5 McFarland standard was prepared from a pure colony inoculated on potato agar and incubated for 7–10 days at 25 °C, according to CLSI M38 (18) and CLSI M44 (19). The antifungals used were as follows: terbinafine (TBF) 1 μg; griseofulvin (GRE) 10 μg; ketoconazole (KTZ) 15 μg; fluconazole (FLZ) 25 μg; itraconazole (ITZ) 10 μg; posaconazole (POS) 5 μg disc (Becton Dickinson, Sparks, MD, USA). *Trichophyton mentagrophytes* ATCC 9533 was used as a quality control strain, using the same materials and methods described previously (20). Both isolates showed sensitivity to all antifungals tested; case 1: TBF (25 mm), GRE (26 mm), KTZ (28 mm), ITZ (30 mm), and POS (36 mm); case 2: TBF (24 mm), GRE (24 mm), KTZ (32 mm), ITZ (30 mm), and POS (36 mm). Fluconazole showed an inhibition diameter of 0 (<14 mm) and was therefore considered resistant in both cases.

## Treatment and resolution

Case 1: After completing the prescribed clotrimazole treatment, the patient returned for a follow-up appointment on day +21 (March 02). The dermatological examination revealed a partial reduction of the lesions, so systemic treatment with itraconazole 5 mg/kg SID (Laboratorio Chile, Ñuñoa, Chile) was prescribed in a pulse regimen of 2 weeks of treatment followed by 1 week of rest for 2 months. On day +81 (May 06), clinical cure was confirmed with complete absence of all lesions (Figure 1C); however, the hair is still observed to be in the process of growing.15 days later, mycological cure was confirmed.

Case 2: Following the consultation, treatment was continued with oral terbinafine 30 mg/kg once daily in pulsed therapy, alternating 14 days of treatment with 7 days of rest in successive cycles until a total of 2 months was completed. On day +30, the patient attended a follow-up appointment, where a significant reduction in the lesion (2–2.5 cm in diameter) and good tolerance to the treatment were observed. Therefore, treatment was continued until its completion on day +60 (October 24). At that time, the absence of the circumscribed lesion on the chin and hair regrowth in the entire area were observed, indicating clinical cure (Figure 2C). 15 days later, a follow-up appointment was scheduled to obtain a new sample (21) and confirm mycological cure.

In both cases, liver function was evaluated with a biochemical profile every 30 days. Additionally, environmental management was prescribed, including daily cleaning and disinfection of surfaces with 0.5% bleach.

## Discussion

*Microsporum canis* is a dermatophyte widely recognized for its zoonotic potential, particularly in households with high levels of human-animal interaction (2–5, 22, 23). Epidemiology highlights the role of cats and dogs as symptomatic and asymptomatic reservoirs and disseminators, as well as the risk of transmission in environments with high animal density (3, 22, 23). In the second case described here, the appearance of compatible lesions in the owner and other domestic animals after the adoption of a cat suspected of having dermatophytosis reinforces this epidemiological scenario and underscores the importance of considering these infections as shared events between veterinary and human medicine. This type of household transmission has been documented in family outbreaks associated with *M. canis* (3, 23).

Dermatophytic kerion in companion animals is an unusual presentation of dermatophytosis and a diagnostic challenge due to the variability of its clinical manifestations and its close similarity to granulomatous forms observed in humans, such as Celsi kerion (4, 5, 7–9). In managing this zoonosis, some practitioners opt for empirical treatment with corticosteroids or antibacterial due to its atypical inflammatory presentation. However, exacerbation of the lesion after corticosteroid use is a clear example of tinea incognito, where iatrogenic immunosuppression caused by these drugs masks or worsens an initial fungal infection, hindering definitive diagnosis (24). This situation underscores the need to strengthen early etiological diagnosis and the prudent use of immunosuppressive and antibacterial therapies, fundamental principles within integrated surveillance and rational antimicrobial use programs promoted by the One Health approach (25). The implementation of rapid and confirmatory diagnostic methods, including molecular tools and antifungal susceptibility testing, not only improves individual clinical management but also contributes to reducing the spread of zoonotic agents and the potential selection of resistant strains in shared domestic and clinical settings (26).

*In vitro* tests performed using the diffusion method on the two isolates showed susceptibility to most of the antifungals tested, except for fluconazole. Resistance to fluconazole and griseofulvin has been previously documented in multiple dermatophyte species (20). The diffusion technique is commonly used; however, it does not surpass the broth microdilution technique, recognized as the gold standard for antimicrobial susceptibility testing because it is quantitative, reproducible, and standardized, allowing the determination of the minimum inhibitory concentration (MIC), which is used as the basis for establishing clinical breakpoints (18, 19).

The phylogenetic analysis did not suggest clustering by sampling site or host; however, one of the Chilean sequences differed from the other *M. canis* sequences analyzed. The inclusion of new Chilean isolates could help us better understand the dynamics of this fungus in the country.

The treatments used in these two cases were based on itraconazole and terbinafine in pulse therapy. Although there is limited evidence for alternate-day therapies in animals, other authors have mentioned that they shorten treatment time, reduce adverse effects, and facilitate medication administration by the owner, achieving clinical and mycological cure (27–29), as observed in these two patients. Additionally, environmental management with cleaning and disinfection protocols is necessary to control the spread and persistence of the fungus on surfaces shared with other animals or people living in the same environment (25, 26).

In this context, interdisciplinary collaboration among veterinarians, physicians, microbiologists, and public health specialists is essential for early detection, epidemiological control, and public education regarding emerging zoonotic dermatophytoses.

## Data Availability

The data presented in the study are deposited in the GenBank repository, accession number PV918591 and PX458008.

## Supplementary material

SUPPLEMENTARY FIGURE 1 Phylogenetic tree and geographic location map generated using the neighbor joining method and constructed using MicroReact with the ITS sequences of Microsporum canis from this work (those represented by a star) and those available in Zhou et al. (4).
