# Supplementary material for: Canine dermatophytic kerion caused by Microsporum canis: two case reports
Source: Front Vet Sci. 2026 Aug 11;13:1809436. doi: 10.3389/fvets.2026.1809436 (PMC13503177; doi:10.3389/fvets.2026.1809436)
Supplement: Supplementary file 1 [file Table_1.DOCX]

Supplementary Material

| **Supplementary Table 1.** Information on the sequences used in the phylogenetic analysis. | | | | |
| --- | --- | --- | --- | --- |
| Sequence number | Genbank access code | Country | Host | Source |
| 1 | OQ181051 | China | Human | Zhou et al. [4] |
| 2 | OQ181052 | China | Human | Zhou et al. [4] |
| 3 | OQ181053 | China | Human | Zhou et al. [4] |
| 4 | OQ181054 | China | Human | Zhou et al. [4] |
| 5 | OQ181055 | China | Human | Zhou et al. [4] |
| 6 | OQ181056 | China | Human | Zhou et al. [4] |
| 7 | OQ181057 | China | Human | Zhou et al. [4] |
| 8 | OQ181058 | China | Human | Zhou et al. [4] |
| 9 | OQ181059 | China | Human | Zhou et al. [4] |
| 10 | OQ181060 | China | Human | Zhou et al. [4] |
| 11 | OQ181061 | China | Human | Zhou et al. [4] |
| 12 | OQ181062 | China | Human | Zhou et al. [4] |
| 13 | OQ181063 | China | Human | Zhou et al. [4] |
| 14 | OQ181064 | China | Human | Zhou et al. [4] |
| 15 | OQ181065 | China | Human | Zhou et al. [4] |
| 16 | OQ181066 | China | Human | Zhou et al. [4] |
| 17 | OQ181067 | China | Human | Zhou et al. [4] |
| 18 | OQ181068 | China | Human | Zhou et al. [4] |
| 19 | OQ181069 | China | Human | Zhou et al. [4] |
| 20 | OQ181070 | Italy | Cat | Zhou et al. [4] |
| 21 | OQ181071 | Italy | Cat | Zhou et al. [4] |
| 22 | OQ181072 | Italy | Cat | Zhou et al. [4] |
| 23 | OQ181073 | Italy | Cat | Zhou et al. [4] |
| 24 | OQ181074 | Italy | Cat | Zhou et al. [4] |
| 25 | OQ181075 | Italy | Cat | Zhou et al. [4] |
| 26 | OQ181076 | Italy | Cat | Zhou et al. [4] |
| 27 | OQ181077 | Italy | Cat | Zhou et al. [4] |
| 28 | OQ181078 | Italy | Cat | Zhou et al. [4] |
| 29 | OQ181079 | Italy | Cat | Zhou et al. [4] |
| 30 | OQ181080 | Italy | Cat | Zhou et al. [4] |
| 31 | OQ181081 | Italy | Cat | Zhou et al. [4] |
| 32 | OQ181082 | Italy | Cat | Zhou et al. [4] |
| 33 | OQ181083 | Italy | Cat | Zhou et al. [4] |
| 34 | OQ181084 | Italy | Cat | Zhou et al. [4] |
| 35 | OQ181085 | Italy | Cat | Zhou et al. [4] |
| 36 | OQ181086 | Italy | Dog | Zhou et al. [4] |
| 37 | OQ181087 | Italy | Dog | Zhou et al. [4] |
| 38 | OQ181088 | Italy | Dog | Zhou et al. [4] |
| 39 | OQ181089 | Italy | Dog | Zhou et al. [4] |
| 40 | OQ181090 | Italy | Dog | Zhou et al. [4] |
| 41 | OQ181091 | Italy | Dog | Zhou et al. [4] |
| 42 | OQ181092 | Italy | Rabbit | Zhou et al. [4] |
| 43 | OQ181093 | Italy | Rabbit | Zhou et al. [4] |
| 44 | OQ181094 | Italy | Rabbit | Zhou et al. [4] |
| 45 | OQ181095 | Italy | Rabbit | Zhou et al. [4] |
| 46 | OQ181096 | Italy | Human | Zhou et al. [4] |
| 47 | OQ181097 | Italy | Human | Zhou et al. [4] |
| 48 | OQ181098 | Belgium | Human | Zhou et al. [4] |
| 49 | OQ181099 | Belgium | Human | Zhou et al. [4] |
| 50 | OQ181100 | Belgium | Human | Zhou et al. [4] |
| 51 | OQ181101 | Guinea | Human | Zhou et al. [4] |
| 52 | OQ181102 | Belgium | Human | Zhou et al. [4] |
| 53 | OQ181103 | Belgium | Human | Zhou et al. [4] |
| 54 | OQ181104 | Romania | Human | Zhou et al. [4] |
| 55 | OQ181105 | Belgium | Human | Zhou et al. [4] |
| 56 | OQ181106 | Belgium | Human | Zhou et al. [4] |
| 57 | OQ181107 | Slovenia | Human | Zhou et al. [4] |
| 58 | OQ181108 | Slovenia | Human | Zhou et al. [4] |
| 59 | OQ181109 | Slovenia | Human | Zhou et al. [4] |
| 60 | OQ181110 | Slovenia | Human | Zhou et al. [4] |
| 61 | OQ181111 | Slovenia | Human | Zhou et al. [4] |
| 62 | OQ181112 | Slovenia | Human | Zhou et al. [4] |
| 63 | OQ181113 | Slovenia | Human | Zhou et al. [4] |
| 64 | OQ181114 | Slovenia | Human | Zhou et al. [4] |
| 65 | OQ181115 | Slovenia | Human | Zhou et al. [4] |
| 66 | OQ181116 | Slovenia | Human | Zhou et al. [4] |
| 67 | OQ181117 | Slovenia | Human | Zhou et al. [4] |
| 68 | OQ181118 | Slovenia | Human | Zhou et al. [4] |
| 69 | OQ181119 | Slovenia | Human | Zhou et al. [4] |
| 70 | OQ181120 | Slovenia | Human | Zhou et al. [4] |
| 71 | OQ181121 | Slovenia | Human | Zhou et al. [4] |
| 72 | OQ181122 | Slovenia | Human | Zhou et al. [4] |
| 73 | OQ181123 | Slovenia | Human | Zhou et al. [4] |
| 74 | OQ181124 | Slovenia | Human | Zhou et al. [4] |
| 75 | OQ181125 | Slovenia | Human | Zhou et al. [4] |
| 76 | OQ181126 | Slovenia | Human | Zhou et al. [4] |
| 77 | OQ181127 | Slovenia | Human | Zhou et al. [4] |
| 78 | OQ181128 | Slovenia | Human | Zhou et al. [4] |
| 79 | OQ181129 | Slovenia | Human | Zhou et al. [4] |
| 80 | OQ181130 | Slovenia | Human | Zhou et al. [4] |
| 81 | OQ181131 | Slovenia | Human | Zhou et al. [4] |
| 82 | OQ181132 | Slovenia | Human | Zhou et al. [4] |
| 83 | OQ181133 | Slovenia | Human | Zhou et al. [4] |
| 84 | OQ181134 | Slovenia | Human | Zhou et al. [4] |
| 85 | OQ181135 | USA | Human | Zhou et al. [4] |
| 86 | OQ181136 | USA | Human | Zhou et al. [4] |
| 87 | OQ181137 | USA | Human | Zhou et al. [4] |
| 88 | OQ181138 | USA | Human | Zhou et al. [4] |
| 89 | OQ181139 | USA | Human | Zhou et al. [4] |
| 90 | OQ181140 | USA | Human | Zhou et al. [4] |
| 91 | OQ181141 | USA | Human | Zhou et al. [4] |
| 92 | OQ181142 | USA | Human | Zhou et al. [4] |
| 93 | OQ181143 | USA | Human | Zhou et al. [4] |
| 94 | OQ181144 | USA | Human | Zhou et al. [4] |
| 95 | OQ181145 | USA | Human | Zhou et al. [4] |
| 96 | OQ181146 | USA | Human | Zhou et al. [4] |
| 97 | OQ181147 | USA | Human | Zhou et al. [4] |
| 98 | OQ181148 | USA | Human | Zhou et al. [4] |
| 99 | OQ181149 | USA | Human | Zhou et al. [4] |
| 100 | OQ181150 | Germany | Human | Zhou et al. [4] |
| 101 | OQ181151 | Germany | Human | Zhou et al. [4] |
| 102 | OQ181152 | Germany | Human | Zhou et al. [4] |
| 103 | OQ181153 | Germany | Human | Zhou et al. [4] |
| 104 | OQ181154 | Germany | Human | Zhou et al. [4] |
| 105 | OQ181155 | Germany | Human | Zhou et al. [4] |
| 106 | OQ181156 | Germany | Human | Zhou et al. [4] |
| 107 | OQ181157 | Germany | Human | Zhou et al. [4] |
| 108 | OQ181158 | Germany | Human | Zhou et al. [4] |
| 109 | OQ181159 | Italy | Cheetah (zoological garden) | Zhou et al. [4] |
| 110 | OQ181160 | Italy | Cat | Zhou et al. [4] |
| 111 | OQ181161 | Italy | Cat | Zhou et al. [4] |
| 112 | OQ181162 | Italy | Without information | Zhou et al. [4] |
| 113 | OQ181163 | Italy | Cat | Zhou et al. [4] |
| 114 | OQ181164 | Italy | Dog | Zhou et al. [4] |
| 115 | OQ181165 | Italy | Cat | Zhou et al. [4] |
| 116 | OQ181166 | Italy | Cat | Zhou et al. [4] |
| 117 | OQ181167 | Italy | Cat | Zhou et al. [4] |
| 118 | OQ181168 | Italy | Cat | Zhou et al. [4] |
| 119 | OQ181169 | Italy | Cat | Zhou et al. [4] |
| 120 | OQ181170 | Italy | Cat | Zhou et al. [4] |
| 121 | OQ181171 | Brazil | Human | Zhou et al. [4] |
| 122 | OQ181172 | Brazil | Cat | Zhou et al. [4] |
| 123 | OQ181173 | Brazil | Cat | Zhou et al. [4] |
| 124 | OQ181174 | Brazil | Cat | Zhou et al. [4] |
| 125 | OQ181175 | Brazil | Cat | Zhou et al. [4] |
| 126 | OQ181176 | Brazil | Cat | Zhou et al. [4] |
| 127 | OQ181177 | Brazil | Cat | Zhou et al. [4] |
| 128 | OQ181178 | Brazil | Cat | Zhou et al. [4] |
| 129 | OQ181179 | Brazil | Cat | Zhou et al. [4] |
| 130 | OQ181180 | Brazil | Cat | Zhou et al. [4] |
| 131 | OQ181181 | Brazil | Cat | Zhou et al. [4] |
| 132 | OQ181182 | Brazil | Cat | Zhou et al. [4] |
| 133 | OQ181183 | Brazil | Cat | Zhou et al. [4] |
| 134 | OQ181184 | Japan | Without information | Zhou et al. [4] |
| 135 | PV918591 | Chile | Dog | Present work |
| 136 | PX458008 | Chile | Dog | Present work |
